# Olaparib combined with immunotherapy for treating a patient with liver cancer carrying *BRCA2* germline mutation: A case report

**DOI:** 10.1097/MD.0000000000022312

**Published:** 2020-09-18

**Authors:** Fengjiao Zhao, Yong Zhou, Poshita Kumari Seesaha, Yihong Zhang, Siqin Liu, Xiaoyan Gan, Jun Hu, Yanhong Gu, Xiaofeng Chen

**Affiliations:** a Department of Medical Oncology, the First Affiliated Hospital of Nanjing Medical University; b Department of Medical Oncology, Nanjing Red Cross Hospital, Nanjing; c OrigiMed, Shanghai; d Department of Oncology, Pukou Branch Hospital of Jiangsu Province Hospital (Nanjing Pukou Central Hospital), Nanjing, China.

**Keywords:** *BRCA2* germline mutation, immunotherapy, liver cancer, next-generation sequencing, PARP inhibitor

## Abstract

**Rationale:**

Immunotherapy and targeted therapy have attracted widespread attention in current clinical research, which could be considered as a good therapeutic option for treatment of refractory liver cancer.

**Patient concerns:**

The patient was a 37-year-old man with hepatitis B virus (HBV) infection. He was presented with hepatalgia and discomfort.

**Diagnosis:**

The computed tomography showed multiple intrahepatic masses, indicating primary liver cancer with multiple intrahepatic metastases.

**Interventions:**

After failed transarterial chemoembolization therapy, he was initially treated with immunotherapy pembrolizumab plus angiogenesis inhibitor lenvatinib, and after 3 months of treatment, the condition improved. However, the disease subsequently progressed. The next-generation sequencing identified a *BRCA2* germline mutation in this patient. A poly (ADP-ribose) polymerase inhibitor, olaparib, plus nivolumab therapy was started and achieved stable disease.

**Outcomes:**

The patient achieved stable disease and improvement in hepatalgia for 3 months after the combination treatment of Olaparib and nivolumab.

**Conclusion:**

We identified a *BRCA2* germline mutation in a patient with liver cancer. Our findings could offer an alternative management for patients with liver cancer harboring germline *BRCA2* mutation.

## 1 Introduction

As a malignant tumor, liver cancer ranks sixth in the morbidity rate and fourth in the mortality rate of cancers worldwide in 2018, with approximately 841,000 new cases and 782,000 deaths yearly.^[1]^ According to the histological type, liver cancer is divided into hepatocellular carcinoma (HCC), intrahepatic cholangiocarcinoma, and combined HCC-cholangiocarcinoma (cHCC-CCA). In most high risk areas of liver cancer, such as China and Eastern Africa, chronic hepatitis B virus (HBV) infection and aflatoxin exposure are the key determinants, whereas in other countries such as Japan and Egypt, the predominant cause is likely HCV infection.^[1]^ The 5-year overall survival rate for liver cancer in China is extremely poor due to high recurrence and metastasis rates.^[2]^ Despite the rapid development of medical technology, there is still no curable strategy for patient with liver cancer. Therefore, an effective and personalized systemic treatment option for liver cancer is strongly needed. Here, we reported a HBV infected-patient with liver cancer carrying a *BRCA2* germline mutation. This patient failed both immunotherapy and chemotherapy treatment, and achieved stable disease (SD) after administration of olaparib plus PD-1 inhibitor nivolumab.

## 2 Case presentation

**Figure 1:**
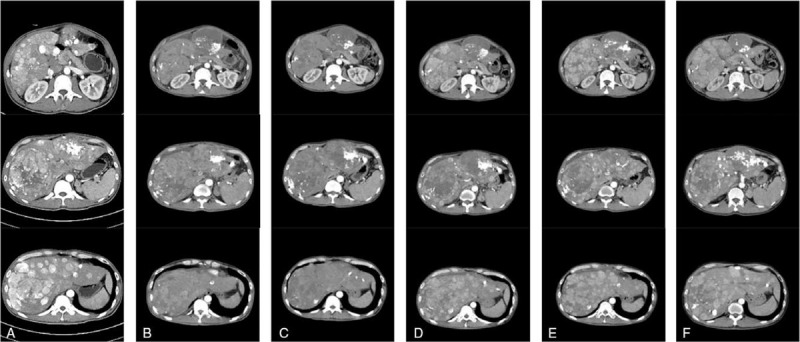
CT scan. (A) Baseline. CT scan showed partial response after lenvatinib plus pembrolizumab treatment for 1 month (B) and 3 months (C). (D) CT scan showed progressive disease after stopping lenvatinib. (E) CT scan showed progressive disease after treatment with nivolumab plus chemotherapy for 1 month. (F) CT scan showed stable disease after treatment with nivolumab plus olaparib for 3 months. CT = Computed tomography.

The patient was a 37-year-old man with HBV infection. He was admitted to a local hospital with hepatalgia and discomfort in December 2017. The computed tomography (CT) showed multiple intrahepatic masses, indicating primary liver cancer with multiple intrahepatic metastases, stage IV. Later, CT showed an enlarged mass during reexamination on January 12, 2018, which suggested potential adrenal metastasis. On January 23, 2018, transarterial chemoembolization (TACE) was started and repeated on March 26, 2018, with documentation of progressive disease (PD) and aggravated hepatalgia according to RECIST 1.1 (Fig. 1A). For further treatment, the patient was referred to our hospital and started lenvatinib (10 mg, po, qd) treatment on May 13, 2018. The levels of glutamic-pyruvic transaminase (123 U/L) and glutamic oxalacetic transaminase (GOT, 230 U/L) increased significantly after 7 days of treatment. Lenvatinib was discontinued and liver protection treatment began. The glutamic-pyruvic transaminase (87 U/L) and GOT (165 U/L) levels decreased and the patient restarted lenvatinib (10 mg, po, qd) treatment on May 25, 2018. From May 23 to September 27, the patient received 7 cycles of immunotherapy with pembrolizumab (100 mg, Q21d). After the second cycle of treatment, the liver lesions were significantly reduced and the hepatalgia basically improved. At the same time, the patient was treated with high-intensity focused ultrasound 43 times from May 26 to September 30. In June and August, CT examination (Fig. 1B, C) showed that the liver lesions reduced and partially disappeared, suggesting partial response evaluated using RECIST 1.1. Due to hepatalgia at the end of September, lenvatinib was withdrawn on September 28. On October 9, CT scanning (Fig. 1D) showed enlarged liver lesions, which indicated PD according to RECIST 1.1. Histopathology of liver biopsy showed unclear structure of the portal area, a different and atypical liver cell nucleus, which indicated liver cancer. The patient received chemotherapy with oxaliplatin (150 mg, d1) plus capecitabine (1000 mg, d1–14) for 2 cycles on October 24, and November 15, respectively. Subsequently, the hepatalgia appeared and the patient turned to nivolumab (140 mg, Q21d) therapy from October 29, 2018. On November 28, CT scanning (Fig. 1E) showed PD according to RECIST 1.1, and chemotherapy was withdrawn.

The tissue biopsy was prepared for next-generation sequencing on October 10, 2018. Mutations of *PIK3CA* E545Q, *JAK1* S703I, *KMT2D* W91^∗^ and *SETD2* E639^∗^, and *AXIN1* rearrangement, as well as a *BRCA2* L2510P germline mutation were identified. On November 30, 2018, the patient started poly (ADP-ribose) polymerase-(PARP) inhibitor olaparib (300 mg, bid) plus nivolumab (140 mg, Q21d) therapy. During a reexamination on March 2, 2019, the hepatalgia basically improved and CT scan showed SD according to RECIST 1.1 (Fig. 1F). No obvious adverse reactions occurred during olaparib plus nivolumab treatment. Unfortunately, during a reexamination on March 29, 2019, CT scan showed PD according to RECIST 1.1. Soon after, the patient stopped olaparib plus nivolumab treatment and taken oral lenvatinib by himself. Subsequently, the patient did not come to the hospital for follow-up treatment. The patient died in home in January, 2020. This study was approved by the Institutional Review Board of the First Affiliated Hospital of Nanjing Medical University. Informed consent for publication of data and images was obtained from the patient included in the study.

## 3 Discussion

This study reports an HBV infection-related liver cancer patient who failed both immunotherapy and chemotherapy treatment. A *BRCA2* L2510P germline mutation was identified in this patient. The patient was then treated with PARP inhibitor olaparib and achieved SD.

The patient initially received TACE therapy. However, after 3 months, the hepatalgia was aggravated and the tumor was progressive. Based on experimental data and clinical data reporting progression of HCC after TACE,^[3]^ it indicated that overexpression of angiogenic and growth factors induced by TACE might be associated with PD after TACE therapy.^[4–6]^ Lenvatinib is an angiogenesis inhibitor that inhibits vascular endothelial growth factor receptor (VEGFR1–3), fibroblast growth factor receptor (FGFR1–4), KIT, and RET.^[7]^ Lenvatinib has been demonstrated to improve outcomes for patients with liver cancer. In the REFLECT phase 3 study of patients with unresectable hepatocellular carcinoma, lenvatinib was confirmed to be noninferior to sorafenib on median survival time (13.6 vs 12.3 months, respectively), and median progression-free survival (PFS) (7.4 vs 3.7 months, respectively).^[8]^

Recently, immunotherapy for cancer has drawn extensive attention in current clinical research. Pembrolizumab (Keytruda), an inhibitor of programmed cell death receptor-1 (PD-1), was approved by the US Food and Drug Administration (FDA) to treat advanced melanoma and nonsmall cell lung cancer patients who do not respond to other treatment. Previous studies demonstrated that PD-1 receptor inhibitors achieved remarkable efficacy in advanced melanoma, nonsmall cell lung cancer, renal cell carcinoma and Hodgkin lymphoma.^[9,10]^ However, pembrolizumab's efficacy in the treatment of advanced liver cancer is still under investigation. Another new drug, nivolumab is a PD-1 blocking antibody, and has been approved by the FDA to treat advanced hepatocellular carcinoma patients.^[11]^ Subsequently, lenvatinib plus immunotherapy with pembrolizumab was started, and a partial response of 3 months was achieved. However, in our patient, hepatalgia was aggravated later, thus lenvatinib was withdrawn and PD appeared. Subsequently, the patient turned to chemotherapy plus immunotherapy with nivolumab for 1 month and achieved PD. Therefore, it was worthwhile to consider switching to an alternative management to clinically control the disease progression.

A *BRCA2* germline mutation in the patient was identified, which suggested involved in disease relapse. Germline mutations in *BRCA1/2* are associated with inheritable risk of breast and ovarian cancer.^[12]^ Germline mutations of *BRCA1/2* were described in 5% to 18% of ovarian cancer and in some other solid malignant tumors including breast cancer, pancreatic cancer, and less frequently in prostate cancer.^[13]^ Few studies have reported the *BRCA1/2* germline mutation in liver cancer. A recent study demonstrated that approximately 2% (7/357) of patients with primary liver cancer carried *BRCA1/2* germline mutations, most of which (5/7) were intrahepatic cholangiocarcinoma.^[14]^

BRCA genes code for proteins that are involved in homologous recombination repair of DNA double-strand breaks. Patients with *BRCA1/2* mutations are sensitive to PARP inhibitors.^[15]^ PARP inhibitors block repair of single-strand breaks, and lead to generation of double-strand breaks in replicating cells. Moreover, defects in BRCA simultaneously prevent homologous recombination, and ultimately lead to genomic instability. The accumulation of DNA damage results in cell cycle arrest and potentially apoptosis.^[16]^ Currently, PARP inhibitor, olaparib, has been approved by the US FDA and European Medicines Agency registration for breast and ovarian cancer with deleterious or suspected deleterious *BRCA1/2* mutations.^[17,18]^ Olaparib showed promising clinical benefits for germline *BRCA1/2* mutated intrahepatic cholangiocarcinoma.^[14]^ It was also demonstrated that combination of olaparib with PD-1 blockade could enhance the antitumor efficacy of olaparib in BRCA1-deficient ovarian cancer.^[19]^ In this case study, the patient was treated with olaparib plus PD-1 inhibitor nivolumab for 3 months and achieved SD and improved hepatalgia. It could provide a reference for patients with liver cancer harboring germline *BRCA1/2* mutations.

In conclusion, since that immunotherapy and targeted therapy have achieved promising clinical effects, more attention should be focused on developing an optimal personalized treatment plan to avoid improper treatment or over-treatment. Mutational analysis by next-generation sequencing may provide important information, which contributes to tailored personalized treatment.

## Author contributions

**Conceptualization:** Fengjiao Zhao, Yong Zhou

**Data curation:** Yihong Zhang, Siqin Liu, Xiaoyan Gan

**Investigation:** Poshita Kumari Seesaha, Jun Hu

**Methodology:** Poshita Kumari Seesaha, Jun Hu, Yihong Zhang

**Validation:** Yanhong Gu, Xiaofeng Chen

**Writing – original draft:** Fengjiao Zhao, Yong Zhou

**Writing – review & editing:** Fengjiao Zhao, Yong Zhou, Poshita Kumari Seesaha, Yihong Zhang, Siqin Liu, Xiaoyan Gan, Jun Hu, Yanhong Gu, Xiaofeng Chen

## Abbreviations

Abbreviations: CT = computed tomography, FDA = Food and Drug Administration, GOT = glutamic oxalacetic transaminase, HBV = hepatitis B virus, HCC = hepatocellular carcinoma, NGS = next-generation sequencing, PARP = poly (ADP-ribose) polymerase, PD = progressive disease, PD-1 = programmed cell death receptor-1, PFS = progression-free survival, SD = stable disease, TACE = transarterial chemoembolization.
